# Ploidy’s Role in Daylily Plant Resilience to Drought Stress Challenges

**DOI:** 10.3390/biology13050289

**Published:** 2024-04-24

**Authors:** Edvinas Misiukevičius, Ingrida Mažeikienė, Vidmantas Stanys

**Affiliations:** Lithuanian Research Centre for Agriculture and Forestry, Institute of Horticulture, Kaunas Street 30, 54333 Babtai, Lithuania; ingrida.mazeikiene@lammc.lt (I.M.); vidmantas.stanys@lammc.lt (V.S.)

**Keywords:** tetraploid plants, plant physiology, adaptation strategies, water deficit, morphological changes

## Abstract

**Simple Summary:**

This research article investigates the ploidy’s impact on daylilies’ (*Hemerocallis* spp.) response to water deficit conditions. By analyzing the morphological and physiological changes in diploid and tetraploid plants under drought stress, this study aims to elucidate the adaptive mechanisms that contribute to their differential tolerance levels. The results reveal distinct responses in the chlorophyll content, flavonoid accumulation, and nitrogen balance between the two ploidy groups, highlighting the superior stress resistance of tetraploid daylilies. The conclusions emphasize the importance of considering ploidy in understanding plant responses to drought stress and suggest the potential for cultivating drought-tolerant varieties to mitigate water scarcity challenges. This study’s insights hold significant value for society by informing strategies for sustainable floriculture, enhancing greenery resilience to climate change, and promoting water-efficient plant breeding practices.

**Abstract:**

This study aimed to understand the differences in the performance of diploid and tetraploid daylily cultivars under water deficit conditions, which are essential indicators of drought tolerance. This research revealed that tetraploid daylilies performed better than diploid varieties in arid conditions due to their enhanced adaptability and resilience to water deficit conditions. The analysis of the results highlighted the need to clarify the specific physiological and molecular mechanisms underlying the enhanced drought tolerance observed in tetraploid plants compared to diploids. This research offers valuable knowledge for improving crop resilience and sustainable floricultural practices in changing environmental conditions. The morphological and physiological parameters were analyzed in 19 diploid and 21 tetraploid daylily cultivars under controlled water deficit conditions, and three drought resistance groups were formed based on the clustering of these parameters. In a high drought resistance cluster, 93.3% tetraploid cultivars were exhibited. This study demonstrates the significance of ploidy in shaping plant responses to drought stress. It emphasizes the importance of studying plant responses to water deficit in landscape horticulture to develop drought-tolerant plants and ensure aspects of climate change.

## 1. Introduction

The impact of climate change on plant physiology is increasingly important as extreme weather events become more frequent and intense [1,2,3]. Water conservation and sustainable management practices must be encouraged within the horticultural industry to adapt to climate change and ensure decorativeness and production sustainability. Understanding the mechanisms that govern plant responses to water deficit and creating drought-tolerant crops is crucial to ensuring food security and sustainable agriculture. Through research on the specific physiological properties that are affected in plants during water deficit periods, scientists can pinpoint potential targets for genetic modification [4] or breeding programs [5], improving crop drought tolerance.

The augmentation of a plant’s ploidy level is known to significantly improve its adaptability to diverse environments. Researchers are increasingly focusing on altering the physiological traits of both diploid and tetraploid plants to develop drought-resistant genotypes. Polyploidy has been found to impact a plant’s habit, xylem structure, and function under water stress [6]. Diploid and tetraploid plants exhibit different physiological responses to water deficits across various crops. For instance, diploid tomato plants exposed to water scarcity showed reduced relative water content (RWC), cell membrane damage, and changes in physiological responses [7]. Intracellular changes during drought have been attributed to the increased removal of reactive oxygen species (ROS) and the accumulation of sugars [8]. Interestingly, tetraploids have been observed to exhibit better performance, less water loss, and less cellular damage compared to diploids when under water stress, implying a higher tolerance to water stress [9].

Daylilies, scientifically known as *Hemerocallis* spp., are among the most popular garden flowers worldwide due to their vibrant blooms and easy maintenance. With various cultivars featuring different ploidy levels, interspecies hybridization is expected in their breeding process [10]. As a result, 100,000 daylily cultivars exist, providing ample opportunity to create beautiful landscapes [11,12]. In the past ten years (2013–2023), 72% of daylilies registered to the American Hemerocallis Society (AHS) were tetraploids. The same tendency has remained in the past five years (2018–2023) of 70% of registered tetraploids [12]. Recent advancements in abiotic stress management have also been made in *Hemerocallis* spp. For instance, *H. fulva* has been shown to mimic drought stress at the transcriptome level [13]. Additionally, a comparative transcriptome analysis of drought moderation between 2× and 4× cv. Trahlyta was conducted in a greenhouse [14]. These studies have revealed complex morphological, physiological, and genetic changes that occur during periods of water deficit. However, the genus *Hemerocallis* remains genetically distant compared to more widely studied plants. As such, the adaptation of daylilies to increasing water deficits at the population level has yet to be extensively studied. While special agronomic measures can be used during drought, selecting and cultivating drought-tolerant varieties in water-deficient areas can provide significant ecological and economic benefits regarding irrigation.

This study aims to analyze the morphological and physiological changes in diploid and tetraploid groups of daylily cultivars under water deficit conditions. By focusing on the factors that contribute to their differential tolerance to drought stress, we hope to gain valuable insights into their adaptation mechanisms. This research will explore the variation in the population and the genotype levels in the morphological and physiological responses of diploid and tetraploid daylilies to water deficit and their ability to tolerate water stress.

## 2. Materials and Methods

### 2.1. Plant Material

**Figure 1:**
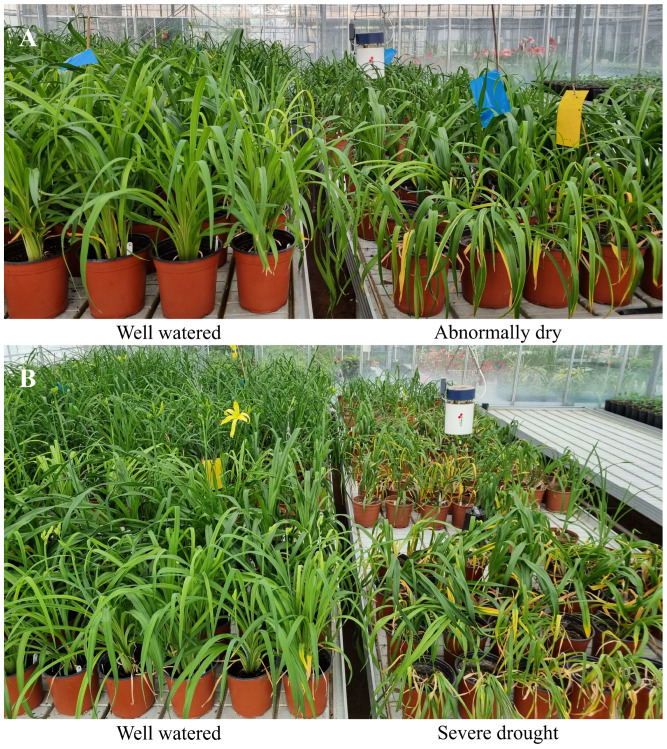
Plants of daylily during modeling drought in greenhouse setting at abnormally dry (**A**) conditions (25% soil moisture) and severe drought (**B**) conditions (10% soil moisture), where plants on the left were well watered (45%). Plants in each group were randomly placed.

There are 19 diploid and 21 tetraploid cultivars and there are different levels of genetic diversity previously described using SSR markers [15] (Supplementary Figure S1). To eliminate transplanting factors, mature daylily divisions were planted in 3-liter containers with a peat and perlite mixture (3:1) and grown for two years. Each genotype included at least six pots divided into two treatment groups: control and drought. All containers with plants were soaked in water before the experiment to provide similar conditions. The soil moisture was measured every three days with the soil moisture meter type HH2 (Delta-T Devices Ltd., Cambridge, UK). The moisture content of the control group of plants was kept at 45% by watering them once a week. The drought treatment plants were not watered, and the measurements of the control and stressed plants were taken simultaneously when the soil moisture levels were constantly 25%—abnormally dry conditions—and 10%—severe drought conditions (Figure 1).

### 2.2. Experimental Location and Design

**Figure 2:**
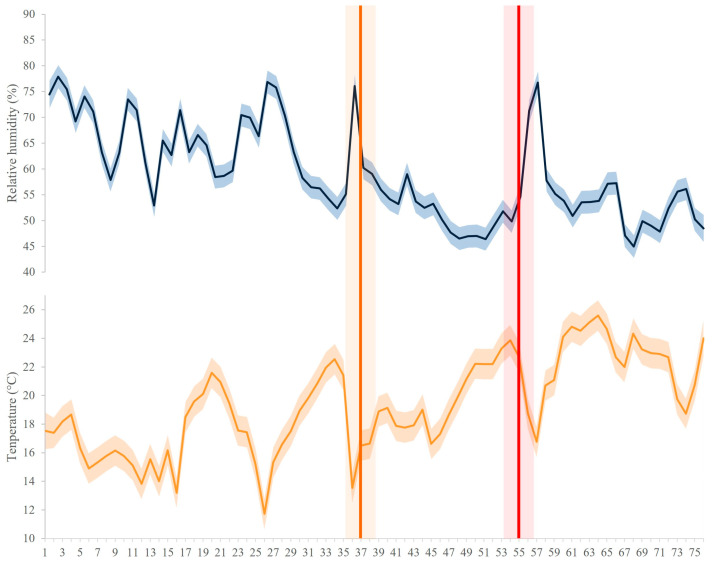
The environmental conditions in the greenhouse during the drought experiment. The temperature (°C) (orange horizontal line) and relative humidity (%) (blue horizontal line) were measured simultaneously every half hour for the entire period. The orange vertical line marks abnormally dry conditions when the soil moisture reached 25%, and the red vertical line marks severe drought conditions, when the soil moisture was 10%. Shaded areas represent ±SE.

Throughout the drought experiment, the temperature and relative humidity were measured in the greenhouse (Figure 2). A digital UNI-T USB datalogger UT330C (Dongguan, China) thermometer placed in the center of the greenhouse was used to measure the temperature, ensuring accurate readings. A digital hygrometer placed at plant height in a representative greenhouse measured the relative humidity. The measured temperature and relative humidity data showed the fluctuation in the parameters where the mean temperature of the experiment was 19.3 °C (ranging from the lowest of 10.7 °C to the highest 26.7 °C) and the relative humidity was 59% (ranging from the lowest of 42.7% to the highest of 80.1%). This extensive monitoring strategy enabled a thorough understanding of the greenhouse’s climatic conditions during the drought experiment.

### 2.3. Data Collection

#### 2.3.1. RWC

A fresh, mature full-length leaf (4–6th from the growing tip) of each treatment plant was collected. The fresh weight (FW) was measured using electronic scales, and each leaf was put in sealed zip-lock bags covered entirely with dH_2_O for 8 h at 4 °C and weighed for the turgid weight (TW). Then, for three days, it was dried at 70 °C, and the dry weight (DW) was measured. The formula calculated the relative water content (RWC), % = (FW − DW)/ (TW − DW) × 100.

#### 2.3.2. ROS

A quantitative analysis of the O_2_ and H_2_O_2_ was performed on the collected six 0.5 cm (about 0.2 in) disks of 3–4 full-length leaves of each plant. For O_2_ detection, the leaf disks were submerged in NBT solution holding sodium azide and NaPO_i_ and infused by vacuum of 100–150 mbar for two times one minute each. After that, the samples were kept in the dark at room temperature (25 °C) for 30 min. The NBT solution was poured out and the samples were submerged with 96% EtOH to wash away the chlorophyll and kept for 16 h. Next, 96% EtOH washing was performed several times until the samples were bleached completely. Then, the EtOH was cleanly poured away, and the samples were lyophilized, weighed, homogenized, and submerged with stock solution holding half 2 M KOH and DMSO. Centrifuged samples for 5 min at room temperature and supernatant absorption were sampled at 580 nm wavelength using a NanoDrop spectrophotometer (Implen, München, Germany). Similarly, the samples for H_2_O_2_ were submerged with a DAB solution holding DAB dye, HCl, Na_2_HPO_4_, and Tween; infiltrated for 5 min in a vacuum of 100–150 mbar; and kept in the dark for 4 h at room temperature. The DAB solution poured out from the samples was switched to EtOH, similarly to O_2_ detection. The lyophilized samples were weighted and submerged with HClO_4_ (0.2 M) solution and centrifuged, and the supernatant absorption was sampled at a 450 nm wavelength. The calculations were made according to Andriūnaitė et al. [16].

#### 2.3.3. Morpho-Physiological Parameters

The plant height was measured for the naturally standing plants, and the leaf length was measured by taking all the leaves and measuring the longest using a ruler. At the end of the experiment, the yellow leaf percentage was analyzed using a ratio of the leaf count and the count of leaves showing decaying tissues. The Dualex 4 Scientific (FORCE-A, Orsay, France) meter was used to take non-destructive measurements of the leaf photosynthetic pigments’ chlorophyll (Chl), flavonol (Flav), and nitrogen balance (NBI) indices.

### 2.4. Drought Stress Response Index

The drought stress response index (DSRI) for individual traits on each genotype at severe drought conditions was calculated using the following formula: DSRI (individual trait) = mean value under drought stress/mean value under well-watered conditions [17]. The computed drought stress response index (CDSRI) for each genotype at severe drought conditions was calculated by combining the DSRI values according to the following formula: CDSRI = (DSRI 1 + DSRI 2 + … + DSRI *n*) − *n*.

### 2.5. Data Analysis

XLSTAT was used to visualize the morphological and physiological data and an analysis of the differences between the categories within the abnormally dry and severe drought treatments with a confidence interval of 95% was performed; Tukey (HSD) was performed using XLSTAT ANOVA. A Gaussian mixture model (GMM) was performed using XLSTAT 2023.3.1. A heatmap and clustering analysis was performed using the pheatmap function in R 4.3.2.

## 3. Results

### 3.1. Drought Stress Affects ROS Components and Leads to Yellow Leaves in Daylily Plants

**Figure 3:**
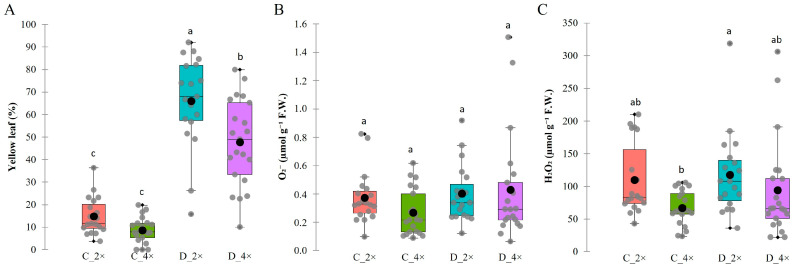
A comparative analysis of *Hemerocallis* spp. in control and stress response diploid (C_2× and D_2×) and tetraploid (C_4× and D_4×) plants: yellow leaf (**A**), reactive oxygen species (ROS) O_2_^−^ (**B**) and H_2_O_2_ (**C**) under well-watered (C) and severe drought (D) conditions. The data are the means (±SE). Within a given treatment, values with the same letter are not significantly different (*p* < 0.05, ANOVA, and Tukey’s). Grey dots represent the mean of individual cultivars in each treatment, and black dots represent the mean of each treatment.

An analysis of the reactive oxygen species (ROS) response to severe drought stress in 2× and 4× daylilies was conducted, and the initial severe drought effects on plant appearance resulted in more abundant yellow leaves (Figure 3). The results of the statistical analysis shown in the boxplot revealed that the 2× and 4× plants showed no significant difference in the yellow leaf percentage under well-watered conditions. However, drought significantly increased the yellow leaf percentage in both ploidy groups (Figure 3A). It was observed that there are significant differences in the yellow leaves in the 2× and 4× plants following prolonged drought conditions. In the diploid plants, 66% of the leaves turned yellow, which is 51% higher than the control.

On the other hand, the 4× plants tended to fare better, with only 48% of the leaves turning yellow, which is 39% higher than the well-watered ones (control). In other words, the 4× plants retained around 18% more leaves than the 2× plants. As the genotype significantly impacts the variation in ROS production, there were no noticeable differences between the ploidy level and drought treatments (Figure 3B,C). Although the plants experienced stress, there were varying degrees of resilience across the groups. Some cultivars were more resistant, while others were more susceptible to water deficit. Based on the data, the 2× plants showed a 28% higher O_2_^−^ rate and a 39% higher H_2_O_2_ rate in well-watered conditions, indicating differences between the two ploidy types. However, under severe drought stress, the 4× plants showed a 61% increase in the O_2_^−^ rate, while the 2× ones remained similar to the well-watered conditions, with only an 8% difference. Similarly, the 4× plants showed a 40% increase in the H_2_O_2_ rate under drought stress, while the 2× ones only showed a 7% increase. 

### 3.2. Effects of Drought Stress on Relative Water Content, Habit, and Leaf Pigment Indices of Daylilies

**Figure 4:**
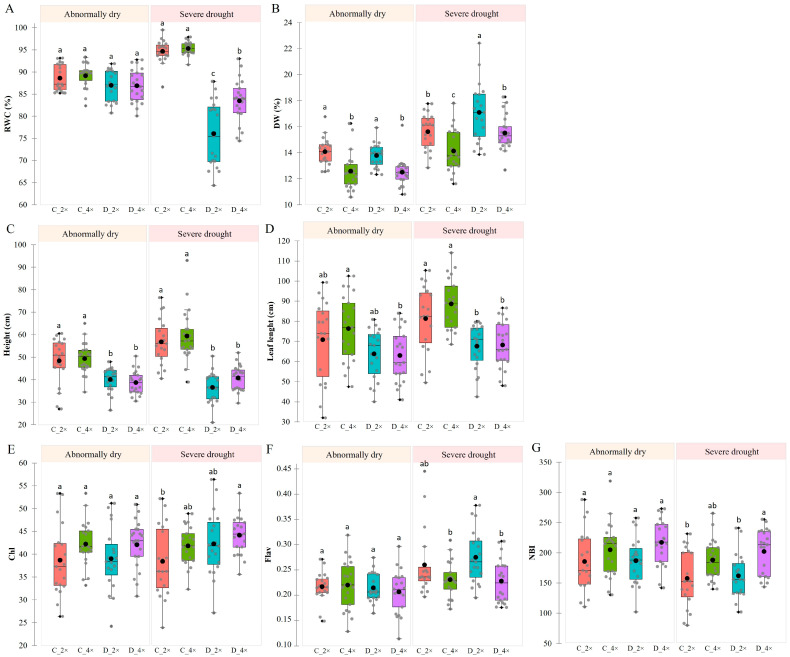
A comparative analysis of *Hemerocallis* spp. in control and stress response diploid (C_2× and D_2×) and tetraploid (C_4× and D_4×) plants: relative water content (RWC) (**A**), dry weight (DW) (**B**), plant height (**C**), leaf length (**D**), leaf pigment indices of chlorophyll (Chl) (**E**), flavonoid (Flav) (**F**), and nitrogen balance (NBI) (**G**); abnormally dry and severe drought conditions. The data are the means (±SE). Within a given treatment, values with the same letter are not significantly different (*p* < 0.05, ANOVA, and Tukey’s). Grey dots represent the mean of individual cultivars in each treatment, and black dots represent the mean of each treatment.

A study assessed the condition of the daylily plants before and during the severe drought. The soil moisture level at the time of evaluation was 25%, categorized as abnormally dry, and 10% as severe drought. The results are shown in boxplots (Figure 4). Seven parameters, including the relative water content (RWC), dry weight (DW), plant height, leaf length, leaf pigment indices of chlorophyll (Chl), flavonoids (Flavs), and nitrogen balance (NBI) of both 2× and 4× daylilies, were analyzed during the assessment. This study was conducted under three conditions: well-watered (control), abnormally dry, and severe drought. During drought stress, the daylily plants exhibited a decrease in RWC and an increase in the dry weight percentage in some instances, as illustrated in Figure 4A,B. The boxplot analysis revealed no changes in the various ploidy or water treatment groups during the abnormally dry conditions, and the RWC percentage ranged from 80 to 95%. In severe drought, the 2× and 4× plants experienced a more significant decrease in the RWC than the well-watered plants in each ploidy group, with a 19% and 12% decrease in the 2× and 4× plants, respectively. The lowest RWC rates during severe drought in the 2× and 4× plants were 64% and 74%, respectively. However, some genotypes in both ploidy groups did not experience water loss. The RWC was 88% in ‘Geltonoji Zvaigzde’ (2×), 86% in ‘Exotic Starfish’ (2×), 93% in ‘Primal Scream’ (4×), 91% in ‘Kloeris-1’ (4×), 89% in ‘Lakelet Wild and Reckless’ (4×), and 88% in ‘Mystical Elf’ (4×). Under well-watered conditions, the diploid daylilies exhibited a 10% higher dry weight than the tetraploids. The abnormally dry conditions did not significantly affect the dry mass of the 2× and 4× plants. However, the severe drought conditions resulted in almost a 10% increase in the dry weight rate in both ploidy groups.

Throughout an 18-day vegetation period, the diploid and tetraploid plants were subjected to a significant reduction in growth due to a water deficit (Figure 4C,D). The plants under stress did not experience any increase in height and lost their shape, whereas their well-watered counterparts grew taller. Under abnormally dry conditions, the height of the 2× and 4× plants decreased by 17% and 22%, respectively. Moreover, the stress modeling indicated that the height of the 2× and 4× plants decreased by 35% and 31%, respectively, during severe drought, indicating a similar trend in height changes for both ploidy groups. The leaf length estimation showed a similar pattern, with the diploid and tetraploid plants experiencing a decrease in length during severe drought. The tetraploids exhibited changes in the leaf length earlier than the 2× plants. Ultimately, both the diploid and tetraploid plants experienced a reduction in height and leaf length during severe drought.

According to Figure 4E, the chlorophyll index (Chl) was statistically equal in the control 2× and 4× plants throughout vegetation, regardless of ploidy. However, when subjected to severe drought, there was a 10% increase in the chlorophyll index of the 2× plants, while a trend of an index increase was observed in the 4× plants at 6%, but no statistical changes were found in either case. On the other hand, the ploidy level did not affect the flavonoids (Flavs) index in the control plants, as shown in Figure 4F. During severe drought stress, however, the 4× plants showed a 17% higher index of flavonoids than the 2× in the same conditions. Similarly, the nitrogen balance index (NBI) showed no significant differences in the controls–well-watered 2× and 4× populations (Figure 4G). However, during severe drought conditions, the 4× plants tended to have higher NBI rates than the 2× plants, with a statistically significant increase of 25%. It is worth noting that diverse genotypes significantly impacted even the most minor changes in each ploidy group.

### 3.3. Drought Response Indexing in Daylilies

**Figure 5:**
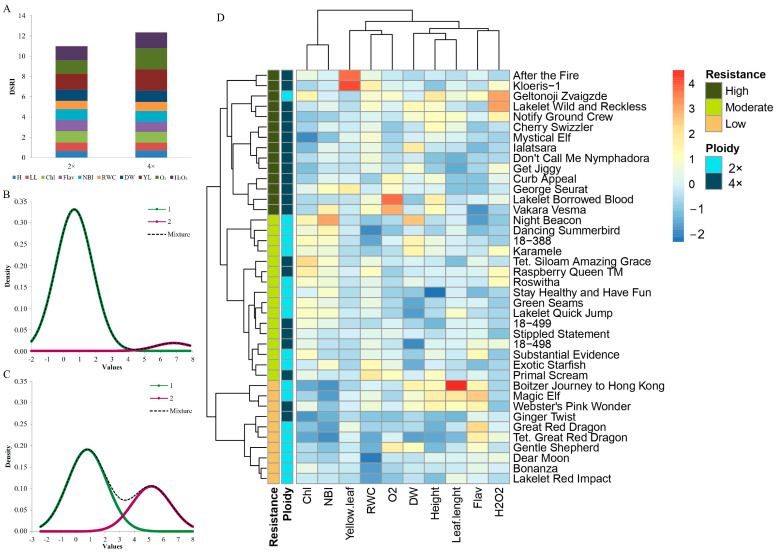
Drought response indexing in daylilies cultivars. Drought stress response index (DSRI) average on each trait given in diploid (2×) and tetraploid (4×) during severe drought (**A**), and Gaussian mixture models (GMM) based on computed drought stress response index (CDSRI) data for 2× (**B**) and 4× (**C**) populations, where the second population indicates highest resistance genotypes from the population. Heatmap and hierarchical clustering (**D**) for morphological and physiological DSRI and CDSRI under severe drought of 2× and 4× daylily genotypes. Clustering analysis of daylily genotypes (left) showed four main groups represent resistance to severe drought based on CDSRI. H—height (cm), LL—leaf length (cm), Chl—chlorophyll index, Flav—flavonoid index, NBI—nitrogen balance index, RWC—relative water content (%), DW—dry weight (%), YL—yellow leaf (%), O_2_—superoxide (µmol g^−1^ F.W.), and H_2_O_2_—hydrogen peroxide (µmol g^−1^ F.W.).

The drought stress response index (DSRI) was used to evaluate the resistant 2× and 4× genotypes in the studied group daylily (Figure 5). This study evaluated the average DSRI values for each trait in the 2× and 4× plant groups to identify the trait’s influence on the resistance rate (as depicted in Figure 5A). The research findings suggest that, in general, the 4× plants exhibited a higher DSRI rate compared to the 2× ones. The primary traits contributing to the 4× resistance rates were a yellow leaf, O_2_, and H_2_O_2_, while the other traits remained consistent. A Gaussian mixture model (GMM) was used to analyze the daylily plants’ CDSRI (cumulative drought stress response index) values. The GMM was performed separately on the 2× and 4× groups of plants (as shown in Figure 5B,C). This analysis divides the cultivars into two distinct populations (green and lilac peaks) based on the data on the response to water deficit. The second population (lilac peak) consisted of genotypes with the highest CDSRI values, indicating high resistance to drought stress. Interestingly, among all the plants analyzed, only one genotype was found in the 2× population, whereas the 4× population contained seven cultivars that showed a distinct resistance to drought stress. A heatmap and hierarchical clustering analysis was performed to assess the resistance groups of the individual genotypes based on the morphological and physiological DSRI values under severe drought conditions (Figure 5D). This study assessed three clusters of resistance groups based on the DSRI value. The clusters were divided into high-, moderate-, and low-resistance groups. The low-resistant cluster contained 80% 2× cultivars. The moderate-resistance cluster had more 4× ploidy cultivars than the 2× one, 62.5% and 37.5%, respectively. However, the high-resistance cluster mainly contained 4× cultivars, 93%, and only one cv. ‘Geltonoji Zvaigzde’ was a 2× ploidy. The study found that 42% of the analyzed 2× genotypes showed low resistance to drought stress, and only 5% showed high resistance. On the other hand, 9% of the 4× genotypes showed low resistance and 62% showed high resistance to drought stress. All the cultivars displayed in the second populations (lilac peaks) of the GMM (Figure 5B,C) were represented in a heatmap resistance cluster.

## 4. Discussion

Polyploidy, or whole genome duplication, is an essential feature of the genome of all eukaryotes [6]. The frequent occurrence of polyploidy indicates the evolutionary advantage of polyploids. No data on naturally occurring tetraploid daylily species have been found. Therefore, polyploidization is considered one of the most important forces of plant evolution. Acquiring a polyploid state can provide competitive advantages for each species and habitat [18]. The development program for tetraploid daylilies began in 1955, led by Robert A. Griesbach and Orville Fay from the USA. They established a technique for inducing tetraploid daylilies using colchicine. This involved exposing germinating seeds to the chemical, as described by Gulia et al. [19]. Polyploidization can lead to the emergence of novel phenotypes or trait variations that were not previously observed in diploid species. These changes can bring several phenotypic benefits, including morphological alterations, physiology, and secondary metabolism. When comparing daylily cultivars with diploid and tetraploid individuals, it has been observed that tetraploid plants tend to have larger flowers, thicker and more compact inflorescences with fewer flowers, broader and longer leaves, and more chlorophyll. Studies conducted by Zhang et al. [20] and Podwyszyńska et al. [21] supported this observation. For instance, polyploids may exhibit enhanced drought tolerance, resistance to pathogens, extended flowering periods, or larger vegetative organs. Such traits are crucial for breeding and can significantly expand the potential applications of polyploids in agriculture. Studies suggest polyploidization can trigger extensive genetic and epigenetic modifications [22]. They include DNA and histone methylation, DNA excision, gene neo- and subfunctionalization, translocations, sequence, and gene expression variations. It is still poorly understood how changes in gene expression contribute to the regulation of complex adaptive traits.

### 4.1. Ploidy Level Affects Plant Morphological and Photosynthetic Pigments’ Response to Drought Stress

Research has shown that 4× plants exhibit a natural resistance to drought [23], which can assert various morphological, physiological, and metabolic changes. Both investigated 2× and 4× daylily cultivar groups experienced reduced plant height and leaf length during severe drought, with the 4× plants exhibiting changes earlier than the 2× ones (Figure 4C,D). Under severe drought, there was an increase in the chlorophyll index of the 2× plants and a trend of increase in the 4× plants, although not statistically significant. The ploidy level did not affect the chlorophyll index in the control–well-watered plants (Figure 4E), showing similar chlorophyll content. Elsalahy and Reckling indicate that during periods of water deficit, changes occur in the chlorophyll system of plants, making it a reliable physiological indicator of stress [24]. Our studies support this claim. During severe drought stress, the 2× daylily plants had significantly more flavonoids than the 4× ones (Figure 4F). This indicates that the diploids were more stressed and began accumulating more flavonoids to help fight drought. According to Walczyk and Hersch-Green’s research [25], a plant’s ploidy level plays a crucial role in determining the nutrient requirements for its growth and development. In our study, while there were no significant differences in the well-watered 2× and 4× populations, the 4× plants tended to have a higher NBI rate than the 2× plants during severe drought, with a statistically significant increase (Figure 4G). During drought stress, the tetraploid daylilies contained more chlorophylls than flavonoids, indicating that a higher NBI corresponded to a more significant presence of chlorophylls than flavonoids. Tetraploid genotypes generally adapt better to water shortage conditions than diploid plants and exhibit larger stomata, higher chlorophyll content, and higher photosynthetic capacity, resulting in a competitive advantage under water-limited conditions [26,27,28]. A previous study with daylily polyploid induction by Misiukevičius and Stanys [29] showed that higher ploidy daylily plants had longer stomata.

### 4.2. Tetraploid Plants Maintain Higher RWC under Drought Stress

The physiological response of daylily plants to drought was influenced by the ploidy level. Both the 2× and 4× plants experienced a significant decrease in RWC during severe drought compared to well-watered conditions. It was noticed that there was a comparable pattern in citrus plants. Tetraploid sour orange (*Citrus aurantium*) plants have better defense mechanisms and maintain higher RWC under drought conditions [30]. However, some genotypes did not experience water loss in our study, even under severe drought conditions (Figure 4A). This demonstrates that the daylily population consists of plants that react differently to drought impacts. The diploid daylilies exhibited a higher dry weight than the 4× ones under well-watered conditions. Severe drought conditions increased the dry weight rate in both ploidy groups (Figure 4B). In the case of bahiagrass (*Paspalum notatum*), tetraploid plants maintain their reproductive performance even under drought conditions due to less starch degradation and higher levels of total phenol content, total soluble sugars, and proline [31]. Additionally, in the *Jasione maritima*, tetraploids show better tolerance to water deficit [9]. A reduction in RWC indicates a water deficit in the plant and is associated with decreased turgor pressure in the plant cells.

During drought stress, the dry weight can be affected by the presence of larger cells of tetraploids. Barceló-Anguiano et al. [32] state that 4× mango trees have larger chloroplasts, mesophyll cells, and stomatal guard cells, resulting in higher leaf elasticity and reduced dehydration rates. As a result, larger tetraploid cells will have less dry mass than diploids. In our study, it was confirmed with a higher dry weight in the 2× than the 4× plants (Figure 4B). Prolonged drought stress reduces the relative water content in pansies, leading to increased dry matter and varying water loss depending on drought intensity and duration. The water content, chlorophyll, and antioxidant activity of pansy plants decrease, but they can recuperate after being watered again [33]. The length and severity of drought affect the plant’s dry matter, indicating water loss during stress. It is well known that severe decreases in RWC due to drought conditions can decrease cell expansion and growth, which leads to an increase in dry mass. Interestingly, it has been observed that abnormally dry conditions do not affect the RWC in daylily plants.

The response of the RWC to drought stress in diploid and tetraploid plants has been a subject of interest in various studies. Tetraploid Rangpur lime rootstock exhibited increased drought tolerance through enhanced constitutive root abscisic acid production, leading to higher RWC than diploid rootstock under water deficit conditions [34]. Similarly, naturally occurring autotetraploids in *Poncirus trifoliata* displayed significantly enhanced drought and dehydration tolerance, contributing to higher RWC than diploid progenitors [8]. Additionally, tetraploid *Spathiphyllum* plants maintained better water balance under drought stress, as evidenced by a less harmful leaf water potential and higher RWC than diploid plants [35]. Furthermore, tetraploids of *Lycium ruthenicum* exhibited a superior stress resistance phenotype under severe drought stress, with increased ABA content and higher RWC than diploids [36]. Lastly, tetraploid fig tree plants demonstrated higher in vitro water stress tolerance, potentially associated with higher RWC than diploid control plants [27]. When subjected to drought stress, autotetraploid apple plants showed higher RWC and chlorophyll fluorescence parameters than diploid apple plants [37]. These findings collectively suggest tetraploid plants generally exhibit enhanced drought tolerance and maintain higher RWC than diploid plants.

### 4.3. Mechanisms for Tetraploid Plant Adaptation to Drought Tolerance

**Figure 6:**
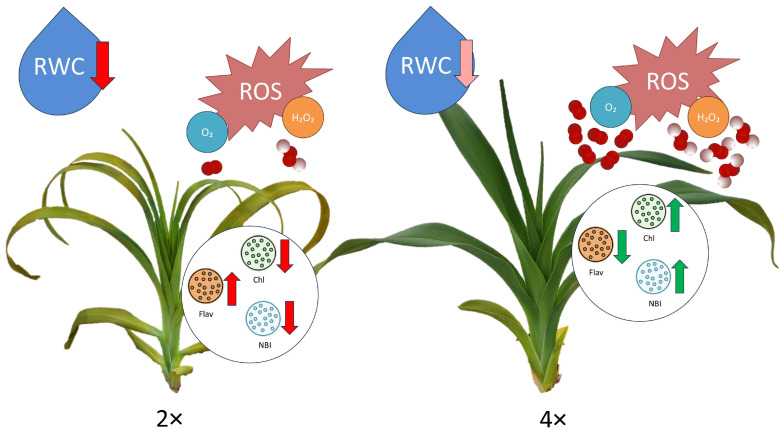
Modeling of strategies to adjust to drought in daylilies. Schemas are based on data comparative analysis of reactive oxygen species (ROS) accumulation, relative water content (RWC), photosynthetic pigments (Chl—chlorophyll content, Flav—flavonoid content, and NBI—nitrogen balance index), plant height, leaf length, and yellowing leaves in diploid (2×) and tetraploid (4×) plants under severe drought stress. The intensity of the RWC downward arrow color indicates the severity of changes, whereas red indicates more significant RWC loss. The orientation of the photosynthetic pigment arrows shows a tendency to increase or decrease, where color indicates whether it has a positive (green) or negative (red) effect on the plant.

Plants exhibit several strategies to adjust to drought. Some plants hasten the flowering and seed maturation process to facilitate self-propagation and prolong their lifespan. Drought escape plants have mechanisms to accelerate their life cycle in response to drought conditions [38]. Other plants undergo the reduction in above-ground components to shield their roots from the adverse effects of drought stress. This reduction in above-ground biomass helps plants conserve water and energy, redirecting resources toward root growth and development to enhance water uptake efficiency and improve drought tolerance [39]. In our study, the 4× daylily plants overall looked better and tended to have greener leaves and higher RWC rates than the 2× ones (Figure 6). The modeling of strategies to adapt to drought in daylilies involved a comparative analysis of the ROS accumulation, RWC, and photosynthetic pigments in the diploid and tetraploid plants under severe drought stress while observing factors, such as plant height, leaf length, and yellowing leaves. Plants exhibit complex regulatory mechanisms in response to drought stress, which involves long-distance signaling from roots to shoots, metabolic regulation at both cellular and whole-plant levels, and ABA synthesis and transport [40]. Polyploidization is known to positively impact the morphology of tetraploid barley by increasing photosynthetic pigments and improving photosynthetic capacity. It also induces changes in the transcriptome, ultimately contributing to plant performance and enhanced photosynthesis [41].

Autotetraploid daylilies exhibit distinct gene expression patterns and responses to drought stress compared to diploid plants, indicating the potential benefits of chromosome doubling in enhancing molecular mechanisms for tolerance to abiotic stress [14]. Differences in the physiological traits, RNA-seq, and secondary metabolome analysis were revealed between the triploid and diploid plants under drought stress, indicating distinct morphological responses based on ploidy level [42]. Severe drought stress resulted in more abundant yellow leaves in the diploid than in the tetraploid daylilies. Differences in ROS production were observed between the two ploidy types under both well-watered and drought stress conditions (Figure 3). The mechanisms underlying this enhanced drought tolerance include increased abscisic acid production, enhanced ROS scavenging, sugar accumulation, and reinforced hormonal, physiological, and biochemical defensive systems in tetraploid plants. ROS play a significant role in plant abiotic stress, including drought stress, and their differential responses in diploid and tetraploid plants need to be evaluated [43]. In our study, we observed a tendency of a greater increase in the H_2_O_2_ and O_2_ rates in the 4× daylily plants than in the 2× ones under drought conditions. Increased ROS levels in 4× plants can trigger signaling pathways that activate a protective stress response and enhance plant tolerance to drought stress [8,14]. *Populus euphratica*, when exposed to gradual soil water depletion, shows reversible changes in gene expression, protein profiles, ecophysiology, and growth performance. Signaling molecules like hydrogen peroxide (H_2_O_2_) and nitric oxide are believed to be essential in mediating the plant’s responses to environmental stimuli [44]. Autotetraploids exhibit enhanced ROS scavenging, a pre-activated ABA response, and differential expression of miRNAs in response to drought stress based on the ploidy level, emphasizing the importance of ROS in plant adaptation to drought stress [8,45,46]. The combination of physiological and molecular data indicates that polyploidization can enhance drought resistance in ‘Hanfu’ and ‘Gala’ apple cultivars [37]. Tetraploid *Solanum betaceum* plants exhibited higher proline accumulation under water stress, indicating better osmoregulation and stress signaling mechanisms than diploid plants [47].

### 4.4. Drought Stress Response

The tetraploids generally exhibited a higher DSRI rate than the diploids under severe drought conditions (Figure 5D). The analysis using a Gaussian mixture model (GMM) for CDSRI values revealed distinct populations based on cultivars, with more 4× genotypes showing resistance to drought stress than the 2× ones (Figure 5B,C). DSRI and CDSRI values were used to evaluate cotton genotypes by estimating physiological and morphological responses to drought stress, showing genetic variability in root parameters [17]. This suggests that daylilies, as perennial plants, have water reserves in their roots that cope with stress way better than other plants. The ploidy level plays a role in the plant’s physiological response to drought, with 4× plants generally exhibiting better adaptation and tolerance. Recent studies show the morphological and physiological responses of 2× and 4× cultivars to drought stress. For instance, triploid aspen had distinct morphological responses such as larger and greener leaves, higher chlorophyll content and leaf mass, and greater stomatal size and stomatal conductance compared to diploids [48]. Drought-tolerant species use water more efficiently, maintaining leaf RWC within narrow limits [49]. These tendencies highlight the varying responses of 2× and 4× daylilies to drought stress, encompassing both morphological and physiological aspects alongside the DSRI and CDSRI findings, which provide insights into the plants’ resistance and emphasize the importance of genotype and environmental conditions in shaping plant resilience and physiological parameters.

## 5. Conclusions

The findings of the studies suggest that ploidy impacts the response of daylilies to drought stress. Tetraploid plants tend to fare better than diploid plants, retaining more leaves and exhibiting a higher stress resistance. However, diploid and tetraploid plants experience a decrease in relative water content and growth during severe drought. It was observed that abnormally dry conditions did not significantly affect the dry mass of both ploidy groups, but severe drought conditions increased the dry weight rate. The chlorophyll index was statistically equal in the control diploid and tetraploid plants throughout vegetation, regardless of the ploidy level. However, when subjected to severe drought, there was a 10% decrease in the chlorophyll index in the diploid plants compared to only a 5% decrease in the tetraploid plants. Even though there was a strong genotype-specific reaction to drought, the tetraploids tended to be more resistant to drought. Overall, the findings highlight the importance of considering ploidy in understanding the effects of drought stress on plant growth and physiology.

## Data Availability

The raw data supporting the conclusions of this article will be made available by the authors on request. The data are not publicly available due to the primary data are contained within the article. Due to the complexity and quantity of datasets, raw data will be provided for scientific purposes on request.

## Supplementary Materials

The following supporting information can be downloaded at: https://www.mdpi.com/article/10.3390/biology13050289/s1, Figure S1: Dendrogram showing relationships among 19 diploid and 21 tetraploid daylily genotypes based on DICE genetic distance matrix obtained by SSR [15]. Blue numbers below branches indicate bootstrap values. Pale brown—diploids; dark blue—tetraploids.
