# Supplementary figures and images for: Ploidy’s Role in Daylily Plant Resilience to Drought Stress Challenges
Source: Biology (Basel). 2024 Apr 24;13(5):289. doi: 10.3390/biology13050289 (PMC11117801; doi:10.3390/biology13050289)

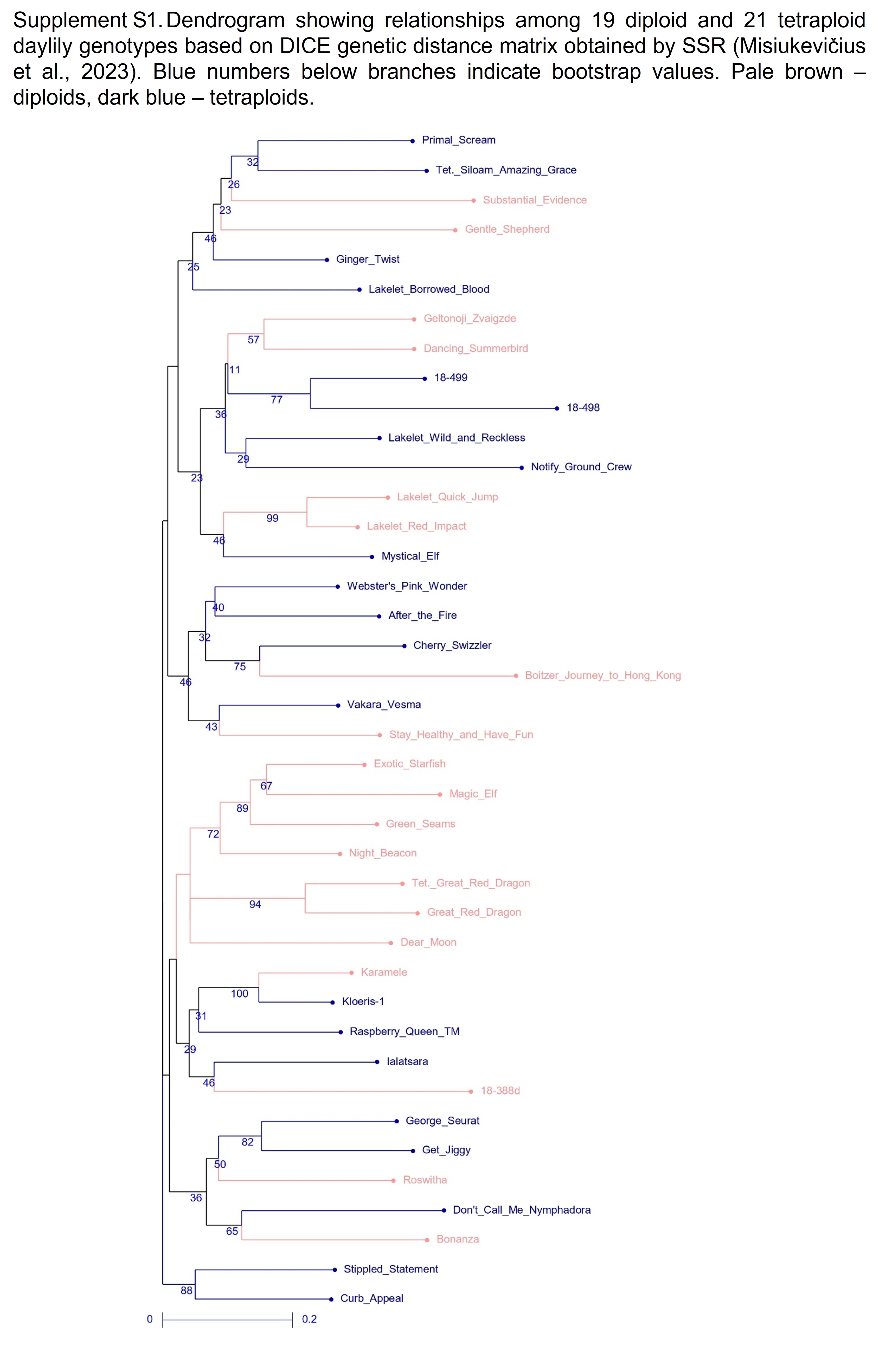

Supplement: Supplementary file 1 [file biology-13-00289-s001.zip › biology-2975246-supplementary.jpg]
